# Eosinophilic Esophagitis Secondary to Sublingual Immunotherapy Using the Aeroallergen Timothy Grass Pollen Allergy Extract: A Case Report

**DOI:** 10.7759/cureus.89867

**Published:** 2025-08-12

**Authors:** Ashwin Agrawal, Jonathan E Teitelbaum, Wendy Shertz-Dipietro, Petro Vavrukh

**Affiliations:** 1 Department of Pediatric Gastroenterology, RWJBarnabas Health, Long Branch, USA; 2 Department of Pathology, Monmouth Medical Center, Long Branch, USA

**Keywords:** dsyphagia, eosinophilic esophagitis (eoe), oral immunotherapy, sublingual immunotherapy, timothy grass pollen

## Abstract

Eosinophilic esophagitis results in a chronic immune-mediated condition of the esophagus characterized by esophageal dysfunction and eosinophil-predominant inflammation. While food-based oral immunity therapy (OIT) is a recognized trigger for eosinophilic esophagitis, the impact of sublingual immunotherapy (SLIT) for aeroallergens is less well established. We present an unusual case of eosinophilic esophagitis in a 14-year-old male induced by SLIT and resolved after discontinuing SLIT with Timothy grass pollen allergy extract (GRASTEK®, ALK-Abelló, Inc., Bedminster, USA), which, to our knowledge, has not been previously reported.

## Introduction

Eosinophilic esophagitis (EoE) results in a chronic immune-mediated condition of the esophagus characterized by esophageal dysfunction and eosinophil-predominant inflammation. It is more common in children with atopic dermatitis, asthma, and food allergies [1]. In children, EoE often presents as dysphagia and is diagnosed by upper endoscopy with esophageal biopsies, which show greater than or equal to 15 eosinophils per high-powered field (HPF) [1]. There are multiple treatment modalities, including using proton pump inhibitors, swallowed steroids, biologic therapy, and avoidance of specific allergens [1]. 

While food-based oral immunity therapy (OIT) is a recognized trigger for EoE, the impact of sublingual immunotherapy (SLIT) for aeroallergens is less well established [2]. We present a case of EoE induced by SLIT and improved after discontinuing SLIT using Timothy grass pollen allergy extract (Grastek®, ALK-Abelló, Inc., Bedminster, USA). A modified version of this case was previously presented as a poster for the 2024 Annual Meeting of the North American Society of Pediatric Gastroenterology, Hepatology, and Nutrition [3].

## Case presentation

A 14-year-old male with a history of environmental allergies presented with heartburn and dysphagia in January 2022. Upper endoscopy was performed using the provider's standard biopsy protocol for concerns of dysphagia, which includes taking at least two biopsies from the distal, mid, and proximal esophagus. Biopsies are placed in the same formaldehyde solution container and provided to pathology for review. Upper endoscopy (esophagogastroduodenoscopy - EGD) revealed an Eosinophilic Esophagitis Endoscopic Reference Score (EoE-EREFS) of 2 points out of 10 points, and esophageal biopsies revealed 31 eosinophils per high-powered field (HPF) (Table 1). He was started on omeprazole 20 mg twice a day.

**Table 1:** Endoscopic and pathologic features from the esophagus during upper endoscopies. Edema, rings, exudate, longitudinal furrows, and stricture are based on grading by the Eosinophilic Esophagitis Endoscopic Reference Score (EoE-EREFS) [1]. SLIT: sublingual immunotherapy

	January 2022	July 2022	August 2023	December 2023
Edema	Grade 1	Grade 0	Grade 1	Grade 0
Rings	Grade 0	Grade 0	Grade 0	Grade 0
Exudate	Grade 0	Grade 0	Grade 0	Grade 0
Longitudinal furrows	Grade 1	Grade 1	Grade 1	Grade 1
Stricture	Grade 0	Grade 0	Grade 0	Grade 0
Peek Eosinophil count per high-powered field	31	23	45	1-2
Eosinophilic micro-abscess	Present	Negative	Negative	Negative
Superficial layering of Eosinophils	Present	Negative	Negative	Negative
Subepithelial fibrosis	Indeterminate	Negative	Mild	Indeterminate
Basal zone hyperplasia	Present	Present	Present	Present
Surface epithelial alteration	Negative	Present	Present	Negative
Dyskeratotic epithelial cells	Negative	Negative	Negative	Negative
Treatment at the time of endoscopy	No treatment	Omeprazole 20 mg twice a day	Omeprazole 20 mg twice a day. Concurrent SLIT using Timothy grass pollen extract	Omeprazole 20 mg twice a day. Discontinued SLIT using Timothy grass pollen extract after the last endoscopy

By July 2022, symptoms had improved, with rare heartburn and no dysphagia. Repeat EGD showed EoE-EREFS score of 1 point out of 10 points (Table 1). Endoluminal functional lumen imaging probe (Endoflip®, Medtronic, Minneapolis, USA) was used to further investigate the esophagus. Our standard approach to Endoflip® for EoE is that the endoluminal balloon is positioned across the lower esophageal sphincter with two sensors within the gastric lumen. The balloon is inflated at 10 cc increments and held at each for 60 seconds. The balloon is inflated until the pressure measurement reaches 20 mmHg or the endoluminal balloon volume reaches 60 mL, whichever occurs first. Observation is made for the presence of retrograde anterior contractions and areas of persistent narrowing. 

The Endoflip® in July 2022 showed normal distensibility but poor contractility. Histopathology revealed 23 eosinophils per HPF. The Index of Severity for EoE (I-SEE) score was consistent with mild disease. Given clinical improvement, shared decision making with the family supported continuation of omeprazole 20 mg twice daily.

In August of 2023, the patient developed generalized abdominal pain and nausea of one month’s duration. Given new abdominal complaints and prior esophageal eosinophilia, a repeat endoscopy to assess for mucosal healing was performed. Endoscopy revealed an EoE-EREFS score of 2 points out of 10 points (Table 1). Histopathology revealed a worsening eosinophilia with 45 eosinophils per HPF. Endoflip® revealed normal distensibility but continued diminished contractility. Discussion with family revealed that two months prior, in June 2023, he was started on Grastek® by his allergist as part of SLIT for grass pollen allergy. Given the worsening of his esophageal eosinophilia on SLIT therapy, the patient was recommended to discontinue Grastek®.

**Figure 1:**
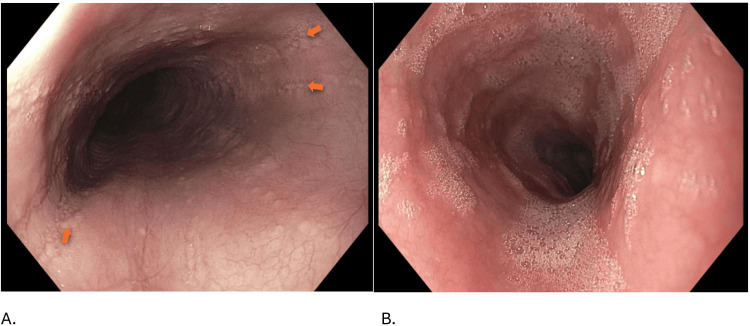
Endoscopic images of the patient. A. The esophagus in August 2023. The orange arrows represent where longitudinal furrows were seen. B. Improvement in the esophagus in December 2023.

**Figure 2:**
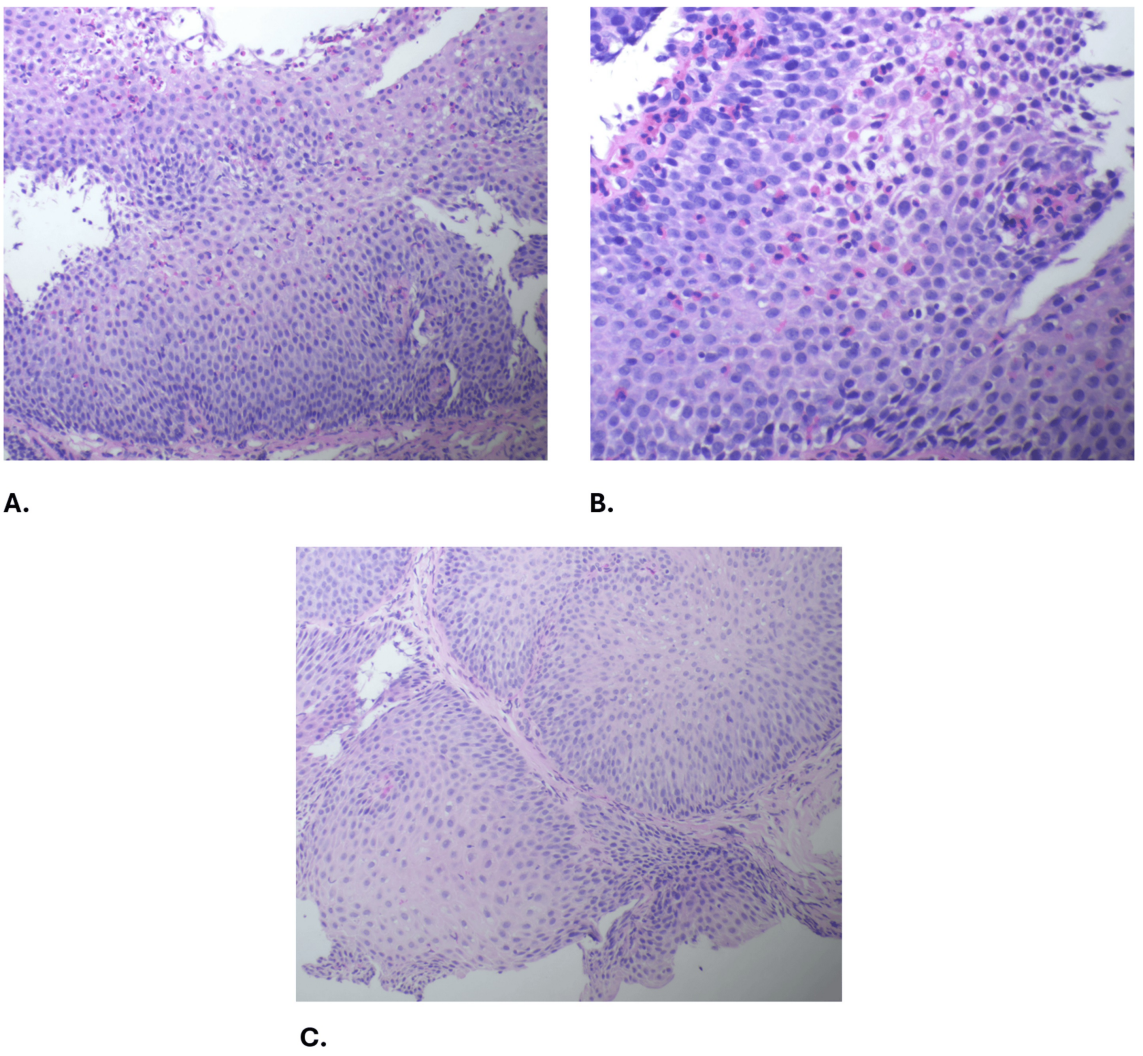
Histopathologic presentation before and after treatment. Histopathologic presentation before (A and B) and after (C) treatment. A. Hematoxylin and eosin stain, 200x magnification. Basal zone hyperplasia with relatively less expansion of the mucosal thickness. Eosinophils are numerous in the superficial layers of the epithelium. B. Hematoxylin and eosin stain, 400x magnification. High-power magnification shows an esophageal biopsy with numerous eosinophils exhibiting superficial distribution (peak eosinophil count 45 eosinophils per high power field, eosinophil degranulation, and intercellular edema. C. Hematoxylin and eosin stain, 200x magnification. Distal esophagus squamous mucosa with mild basal hyperplasia and associated scattered lymphocytes and rare eosinophils.

Repeat EGD four months later revealed an EoE-EREFS score of 1 point out of 10 points (Table 1). Endoflip® revealed normal distensibility and normal contractility. Endoscopic improvement is seen in Figure 1. Histopathology revealed resolution of EoE with 1-2 eosinophils per HPF (Figure 2).

## Discussion

This case highlights a rare but important adverse effect of SLIT with environmental allergens. While food-based OIT is an established trigger for EoE, with reported incidence rates between 1-3.2%, the association between SLIT for aeroallergens and EOE is less well characterized [2,4,5].

EoE is a chronic immune-mediated esophageal disease characterized by symptoms related to esophageal dysfunction and histologically by eosinophilic inflammation defined as greater than or equal to 15 eosinophils per high-power field on esophageal biopsy [1]. It is a leading cause of dysphagia in children and young adults [1]. The pathophysiology of EoE is thought to be multifactorial and not yet fully known. It is thought to result from a complex interplay between genetic predisposition, environmental exposures, and impaired barrier function [6]. EoE is associated with other atopic diseases such as asthma, allergic rhinitis, eczema, and food allergies. Seasonal variation in EoE activity and symptom exacerbation during high pollen months have been reported, supporting the role of aeroallergens as contributors to disease activity [6].

Several case reports have documented the onset of EoE during or after initiation of SLIT or OIT for pollens and other environmental allergens [6-10]. This includes a pediatric patient who developed EoE due to SLIT for pollen [6] and a pediatric patient who developed EoE with the use of SLIT for *Alternaria* [7]. Another case demonstrated EoE caused by a combination of environmental oral immunotherapy using drops of Bermuda/rye grass pollen, a mixture of tree pollens, and dust mite extract, which resolved with discontinuation of this therapy [8]. One case also revealed an adult patient who developed EoE after SLIT with *Dermatophagoidespteronyssinus* and *Dermatophagoides farinae *[9]. One case report showed EoE recurrence in a pediatric patient taking SLIT using fivetypes of grass pollen [10]. However, to our knowledge, EoE in response to Timothy grass pollen allergy extract SLIT (Grastek®) has not previously been reported. 

The pathophysiology of how environmental allergens cause EoE is not known. However, the 2023 International Consensus Statement on Allergy and Rhinology lists EoE as a contraindication for SLIT [4]. Furthermore, a clinical practice guideline in 2024 from the American Academy of Otolaryngology-Head and Neck Surgery specifically advises not to initiate SLIT in patients with known EoE [11]. Moreover, the Grastek® product label identifies EoE as a contraindication, citing its European counterpart, Grazax® (ALK-Abelló, Inc., Bedminster, USA), as having caused EoE, though no referenced cases are provided [12].

Our patient’s histologic improvement following cessation of SLIT strongly supports a causal relationship. Multiple reports, including this one, have shown that withdrawal of SLIT or OIT leads to resolution of EoE when it is the causative agent [2, 6-10].

As more allergists and pediatricians prescribe immunotherapies for food and environmental allergens, this case underscores the importance of counseling patients and families about the risk of developing EoE. In addition, if OIT/SLIT is started in a patient with known EoE, close monitoring for worsening of EoE is required. Allergists and pediatricians should ask the patient if they have been diagnosed with EoE prior to prescribing OIT/SLIT. If the patient has EoE, the provider considering OIT/SLIT should consult with the gastroenterologist prior to starting therapy due to the high risk of worsening of EoE with OIT/SLIT. Our case highlights the importance of good clinical history, especially when EoE worsens symptomatically or asymptotically, and assessing other therapies the child may be on, including SLIT, which may have worsened the EoE.

## Conclusions

We present an unusual case of EoE in the pediatric population that was associated with the use of sublingual immunotherapy using Timothy grass pollen allergy extract (Grastek®). Children on oral or sublingual immunotherapy for environmental allergens should be monitored for signs and symptoms of EoE.

Allergists and pediatricians should ask the patient if they have been diagnosed with EoE prior to prescribing OIT/SLIT. If the patient has EoE, the provider considering OIT/SLIT should consult with the gastroenterologist prior to starting therapy due to the high risk of worsening of EoE with OIT/SLIT. If EoE develops, oral immunotherapy should be discontinued.
